# Metabarcoding free‐living marine nematodes using curated 18S and CO1 reference sequence databases for species‐level taxonomic assignments

**DOI:** 10.1002/ece3.4814

**Published:** 2019-01-22

**Authors:** Lara Macheriotou, Katja Guilini, Tania Nara Bezerra, Bjorn Tytgat, Dinh Tu Nguyen, Thi Xuan Phuong Nguyen, Febe Noppe, Maickel Armenteros, Fehmi Boufahja, Annelien Rigaux, Ann Vanreusel, Sofie Derycke

**Affiliations:** ^1^ Marine Biology Research Group, Department of Biology Ghent University Ghent Belgium; ^2^ Department of Nematology, Institute of Ecology and Biological Resources Vietnam Academy of Science and Technology Hanoi Vietnam; ^3^ Centro de Investigaciones Marinas Universidad de La Habana Habana Cuba; ^4^ Laboratory of Biomonitoring of the Environment (LBE), Faculty of Sciences of Bizerte University of Carthage Carthage Tunisia; ^5^ Aquatic Environment and Quality, Institute for Agricultural and Fisheries Research (ILVO) Oostende Belgium; ^6^ OD Taxonomy and Phylogeny, Royal Belgian Institute of Natural Sciences (RBINS) Brussel Belgium

**Keywords:** metabarcoding, mock community, Nematoda, reference sequence database, UClust, USearch9

## Abstract

High‐throughput sequencing has the potential to describe biological communities with high efficiency yet comprehensive assessment of diversity with species‐level resolution remains one of the most challenging aspects of metabarcoding studies. We investigated the utility of curated ribosomal and mitochondrial nematode reference sequence databases for determining phylum‐specific species‐level clustering thresholds. We compiled 438 ribosomal and 290 mitochondrial sequences which identified 99% and 94% as the species delineation clustering threshold, respectively. These thresholds were evaluated in HTS data from mock communities containing 39 nematode species as well as environmental samples from Vietnam. We compared the taxonomic description of the mocks generated by two read‐merging and two clustering algorithms and the cluster‐free Dada2 pipeline. Taxonomic assignment with the RDP classifier was assessed under different training sets. Our results showed that 36/39 mock nematode species were identified across the molecular markers (18S: 32, JB2: 19, JB3: 21) in UClust_ref OTUs at their respective clustering thresholds, outperforming UParse_denovo and the commonly used 97% similarity. Dada2 generated the most realistic number of ASVs (18S: 83, JB2: 75, JB3: 82), collectively identifying 30/39 mock species. The ribosomal marker outperformed the mitochondrial markers in terms of species and genus‐level detections for both OTUs and ASVs. The number of taxonomic assignments of OTUs/ASVs was highest when the smallest reference database containing only nematode sequences was used and when sequences were truncated to the respective amplicon length. Overall, OTUs generated more species‐level detections, which were, however, associated with higher error rates compared to ASVs. Genus‐level assignments using ASVs exhibited higher accuracy and lower error rates compared to species‐level assignments, suggesting that this is the most reliable pipeline for rapid assessment of alpha diversity from environmental samples.

## 1 INTRODUCTION

Species identification is an indispensable part of ecological studies and the most basic form of biological data. Traditionally, this has been achieved through the meticulous study of morphological features, a time‐consuming process demanding expert taxonomical knowledge. Currently, metabarcoding using high‐throughput sequencing (HTS) allows for the acquisition of large volumes of sequence data from multiple bulk samples containing numerous individuals, offering a cost‐effective, efficient, and rapid way of assessing the diversity of complex communities. DNA barcode sequences stored in digital repositories (e.g., GenBank, SILVA, BoLD) coupled to morphologically identified voucher specimens expedite species identification by serving as taxonomically assigned references (Hebert, Ratnasingham, & de Waard, 2003).

Operational taxonomic units (OTUs) are most commonly used as a proxy for species in HTS data. OTUs can be generated by heuristic (e.g., USearch9, UClust), hierarchical (e.g., Dotur, Esprit), and/or model‐based (e.g., Crop) clustering of sequence reads in the presence or absence of a user‐defined similarity percentage (Edgar, 2010, 2013; Hao, Jiang, & Chen, 2011; Schloss & Handelsman, 2005; Sun et al., 2009). Clusters are formed on the basis of similarity to a collection of reference sequences (reference‐based), the assemblage of reads present in the sample (denovo) or a combination of both (open‐reference) (Schloss & Westcott, 2011). Heuristic algorithms which implement clustering thresholds are typically preferred over computationally demanding hierarchical methods, with UClust and USearch being two of the most commonly used (Chen, Zhang, Cheng, Zhang, & Zhao H, 2013). Ideally, clustering thresholds should correspond to inter‐ and intraspecific sequence divergence data in order for OTUs to function as a proxy for species. Typically, sequence similarity is set to 97% based on bacterial species delineation via DNA–DNA hybridization (Stackebrandt & Goebel, 1994) yet has been shown to differ between taxonomic groups and molecular markers (Behnke et al., 2011). Recently, clustering‐free approaches such as Dada2 and Deblur are becoming increasingly popular, the former being the recommended strategy in Qiime2 (Amir et al., 2017; Callahan et al., 2016). Dada2 effectively “corrects” reads to yield true biological sequences by applying a “quality‐aware model of Illumina amplicon errors and sample composition is inferred by dividing amplicon reads into partitions consistent with the error model” (Callahan et al., 2016); the performance of amplicon sequence variants (ASVs) for eukaryotes has yet to be addressed. Taxonomy can be assigned to each OTU/ASV using several methods (e.g., alignment, tree, or probability‐based), all of which require reference datasets. The accuracy of taxonomic assignment is thus strongly dependent upon the availability of reference sequences coupled to credible morphological species identifications as well as the algorithm of choice (Holovachov, 2016; Holovachov, Haenel, Bourlat, & Jondelius, 2017; Somervuo et al., 2017). In this respect, the Naïve Bayesian Ribosomal Database Project classifier (RDP) has been shown to yield increased taxonomic assignments and is currently the recommended strategy by Qiime developers (Navas‐Molina et al., 2013; Ritari, Salojärvi, Lahti, & de Vos, 2015).

Free‐living marine nematodes are found in almost every sedimentary environment (Vanreusel et al., 2010) where they are typically the most numerous meiofaunal taxon and essential to ecosystem functioning by facilitating mineralization, nutrient cycling as well as the provision of a high‐quality food source (Bonaglia, Nascimento, Bartoli, Klawonn, & Brüchert, 2014; Leduc, 2009). Morphological identification of nematodes demands the time‐consuming process of compound microscopy while the existing taxonomical knowledge is insufficient for species‐level identification as the majority are undescribed (Appeltans et al., 2012). A high degree of phenotypic plasticity and cryptic speciation further complicates the delineation of species using morphological characteristics exclusively (Derycke et al., 2008, 2005; De Ley et al., 2005). Resultantly, metabarcoding is being adopted to assess nematode diversity using the ribosomal 18S locus which generally provides resolution to genus and higher taxonomic levels but often fails to distinguish between congeneric species (Armenteros et al., 2014; Bik, Sung, et al., 2012; Carugati, Corinaldesi, Dell'Anno, & Danovaro, 2015; Derycke, Ley, et al., 2010; Porazinska, Giblin‐Davis, Esquivel, et al., 2010). Variability in repeat copy number and intragenomic variation generate a consistent pattern with each species containing a few highly abundant OTUs along with a large collection of rare OTUs (“head‐tail” pattern), thereby confounding estimates of diversity (Bik, Porazinska, et al., 2012; Porazinska, Giblin‐Davis, Sung, & Thomas, 2010). The mitochondrial cytochrome oxidase 1 (CO1) has hitherto not been applied for nematode metabarcoding, despite its capacity for species‐level resolution in marine nematodes (Derycke et al., 2005).

Using nematode mock communities, our objectives were to 1/evaluate the utility of OTUs as a proxy to species by calculating marker‐specific clustering thresholds derived from inter‐ and intraspecific p‐distance values from curated Sanger sequences, 2/compare the efficacy of the 18S ribosomal and mitochondrial COI molecular markers in nematode metabarcoding, 3/compare two read‐merging strategies for generating OTUs (Pear/Fastq_mergepairs; the former evaluates all possible paired‐end read overlaps without requiring the target fragment size as input and implements a statistical test for minimizing false‐positive results while the latter computes the optimal ungapped alignment of the overlapping region of the forward and the reverse‐complemented reverse sequence), 4/compare open‐reference and *denovo* heuristic clustering algorithms (UClust*_ref*/USearch9) as well as the clustering‐free Dada2 pipeline at describing species diversity, and 5/determine the effect of reference sequence databases of different size, length, and taxonomic coverage on OTU clustering and taxonomic assignment with RDP. We expected the ribosomal data to yield an inflated number of OTUs due to the aforementioned “head‐tail” pattern (Porazinska, Giblin‐Davis, Sung, et al., 2010) while the mitochondrial data to yield species‐level taxonomic resolution. Finally, the analysis pipeline which generated the most accurate description of the mock communities was applied to an environmental sediment sample from Vietnam in which nematode species have been identified morphologically.

## 2 MATERIALS AND METHODS

### 2.1 Sanger data

We generated 18S and CO1 sequences from nematodes sampled in the equatorial North Pacific, Cuba, Italy (Panarea Island), Papua New Guinea, the Netherlands, Tunisia, and Vietnam. All nematodes were vouchered and identified by taxonomic experts prior to molecular manipulation. Individual specimens were picked from a plastic counting tray using a metal needle, washed three times in sterile Mili‐Q water, mounted on temporary glass slides, and assigned to the lowest taxonomic level using a camera‐equipped microscope while simultaneously photographing morphological features (specimens from Cuba were processed as described in Armenteros et al., 2014). Genomic DNA was extracted from all samples as follows: Each specimen was placed in a 0.5‐ml Eppendorf tube with 20 µl worm lysis buffer (50 mM KCl, 10 mM Tris pH 8.3, 2.5 mM MgCl_2_, 0,45% NP 40 (Tergitol Sigma), and 0,45% Tween 20). DNA extractions were completed by adding 1 µl proteinase K [10 mg/ml] and heating to 65°C for 60 min followed by 95°C for 10 min. Samples were then centrifuged at maximum speed for 1 min and lysates stored at −20°C. DNA extracts were used to amplify a ca. 800 bp 18S ribosomal (V1‐V2) and ca. 396 bp CO1 mitochondrial fragment (I3‐M11) with primers SSU_F_04‐4R and JB3‐JB5, respectively; when these failed, we amplified shorter fragments of the same loci, ca. 360 bp and 340 bp for 18S and CO1 using primers SSU_F_04‐SSU_22_R and JB2‐JB5GED (Armenteros et al., 2014; Blaxter et al., 1998; Derycke et al., 2007, 2005). Sequences coupled to a voucher specimen with reliable species identification were used to calculated intra‐ and interspecific p‐distances: 438 and 290 sequences for 18S and CO1 (“refDBs”) with each species being represented by maximum eight and 22 specimens, respectively (Supporting Information Appendix S4). Sequences were aligned using Muscle and default settings in Geneious® v.9.1.6 (Edgar, 2004; Kearse et al., 2012). Stop codons were manually removed from the CO1 alignment. The software Gblocks© v.0.91b (Castresana Lab, 2002) was used to remove poorly aligned positions and divergent regions of 18S sequences (minimum number of sequences for conserved position: 50%, minimum number of sequences for flank position: 50%, maximum number of contiguous non‐conserved positions: 8, minimum length of block: 5, and gap tolerance: with half). P‐distances were calculated in Mega7.0.18 using default settings and pairwise deletion of gaps/missing data (Kumar, Stecher, & Tamura, 2016). The resulting similarity matrix was imported to ExCaliBar v1.0.0.0 (Ferdowsi University of Mashhad, Iran) for sorting the data into intra‐ and interspecific distances and visualized as histograms in GraphPad Prism v6 (GraphPad Software, La Jolla, CA, USA). Intraspecific values greater than 0.05 were used as an indication of potential cryptic species (Derycke, Vanaverbeke, Rigaux, Backeljau, & Moens, 2010). In these instances, the relevant voucher specimens were double‐checked to verify taxonomic identity and cryptic speciation confirmed when the following three conditions were met: 1/The p‐distance was high for both CO1 and 18S to rule out random sequence divergence of either locus, 2/the relevant specimens came from different regions (divergence through isolation), and 3/the p‐distances showed consistent patterns that divided specimens into clear groups, that is, low values within and high between groups. Cryptic species were indicated as such by appending a letter to the label. Finally, species delimitation distance thresholds were obtained using the ad hoc package in RStudio© v0.99.878, with a maximum relative error (RE) of 0.05 and ambiguous identifications treated as correct (Sonet et al., 2013). This could not be completed for the 18S data as a whole due to the overlap of inter‐ and intra‐specific distances; we therefore performed all aforementioned steps separately on two families from the three best represented orders; Chromadoridae (*n* = 27), Cyatholaimidae (*n* = 38), Oncholaimidae (*n* = 29), Oxystominidae (*n* = 16), Sphaerolaimidae (*n* = 15), and Xyalidae (*n* = 49). A threshold value within 5% RE could not be derived for Chromadoridae, Oncholaimidae, and Xyalidae; for these families, the interval coupled to the lowest possible RE was provided.

### 2.2 Mock communities

DNA extracts from vouchered nematode specimens were pooled to create two replicate mock communities for two experimental treatments: equimolar (A/B) and isovolumetric at 1 µl DNA extract per specimen (C/D). The A/B mocks consisted of 53 DNA extracts representing 39 nematode species (Supporting Information Table S1). A reference sequence for 18S and CO1 was not available for four DNA extracts and thus was not included in mocks C/D as well as the specimen Spilophorella_aberrans_64H6K12 (insufficient extract). A mitochondrial sequence for specimen Halalaimus_sp_BEL_4 was not available. As such, DNA extracts from 53 and 48 specimens belonging to 39 and 35 species were present in mocks A/B and C/D, respectively.

### 2.3 HTS library preparation

The DNA extract concentrations were measured using the Qubit® dsDNA High Sensitivity Assay Kit (Thermo Fisher Scientific, USA) and equimolarly pooled for mocks A/B. The CO1 and 18S loci were amplified using primers JB2‐JB5GED (JB2), JB3‐JB5 (JB3), and SSU_F_04‐SSU_22_R (18S), respectively; these were constructed with Illumina overhang adapters as described in “16S Metagenomic Sequencing Library Preparation.” To minimize and test for the bias generated by multiple PCR cycles toward the most abundant taxa (Suzuki & Giovannoni, 1996), we chose two cycling conditions, 30 and 12 cycles. The four samples were amplified in triplicates with the following PCR conditions: 18S: 95°C 2 min, 12/30× (95°C 1 min, 57°C 45 s, 72°C 1 min), 72°C 10 min; CO1: 95°C 2 min, 12/30× (95°C 1 min, 50°C 45 s, 72°C 3 min), 72°C 10 min. The mix consisted of 8.4 μl PCR‐grade H_2_O, 4 µl Phusion buffer, 4 µl Dye, 0.4 µl dNTP [10 mM], 1 µl forward and reverse primer [10 μM], 0.2 µl Phusion Hot Start II High Fidelity Polymerase (New England BioLabs, USA). The products were run on a 1% agarose electrophoresis gel to confirm fragment length. The 30 cycle libraries were purified using E‐Gel® SizeSelect™ Agarose Gel 2% (Thermo Fisher Scientific) and the 12 cycles with Agencourt AMPure XP beads (Beckman Coulter, USA). The 12 and 30 cycle products were run on the Bioanalyzer 2100 High Sensitivity and 7500 DNA Kit, respectively, to assess the size distribution of PCR fragments. Triplicates were pooled, resulting in a total of 24 libraries, that is, two cycling conditions × three primer pairs × two mock communities × two replicates. Library indexing was completed using the FC131‐1002 NexteraXT Index Kit (Illumina, USA) following the aforementioned Illumina protocol, purified using Agencourt AMPure XP beads, run on 2100 Bioanalyzer DNA 7500 Kit, and quantified using Qubit® dsDNA High Sensitivity Assay Kit. All libraries were pooled at a 10 nM concentration. For eight of the low cycle libraries, we were unable to obtain a Qubit reading; these were pooled at 5 µl per library. Mock libraries as well as libraries of the same loci from three Vietnamese field samples (A2, A3, A4) were sequenced at Edinburgh Genomics on one Illumina MiSeq‐v3 2 × 300 bp paired‐end reads run (https://genomics.ed.ac.uk/).

### 2.4 Bioinformatics

We compared two read‐merging, two clustering algorithms, the Dada2 pipeline and assessed the accuracy of taxonomic assignment with the RDP classifier (Wang, Garrity, Tiedje, Cole, & Al, 2007) given reference databases differing in the number, length, and taxonomic coverage of sequences (pipeline flowchart, Supporting Information Figure S2). Raw data in fastq format were delivered demultiplexed. The low PCR cycle samples failed to yield sufficient reads and were not analyzed. Reads were processed separately for 18S, JB2, and JB3 with the exception of chimera detection in which the JB2/JB3 datasets were combined. Forward and reverse reads were merged and quality filtered using two algorithms: 1/Paired‐End reAd mergeR (PEAR v0.9.10, Zhang, Kobert, Flouri, & Stamatakis, 2014) and 2/Fastq_mergepairs in Usearch9 [v9.2.64_i86linux32 (Edgar, 2010)]. Gene‐specific adapter sequences were truncated from the 5′ and 3′ read ends using Cutadapt (v1.12, Martin, 2011) by firstly trimming the 5′ adapter (anchored) followed by trimming the 3′ adapter (anchored) with untrimmed sequences discarded. Chimeric sequences were removed with the USearch61 algorithms Uchime*_ref* and Uchime*_denovo* in Qiime1, retaining reads only if they were flagged as non‐chimeric by both with the Silva99_refDB_mock and refDB_mock databases for ribosomal and mitochondrial reads, respectively. Stop codons were removed from the CO1 data using custom scripts. Reads were clustered using the cluster_otus command in USearch9 and pick_open*_*reference_otus.py in Qiime1. We compared five reference databases for UClust_*ref* clustering and taxonomic assignment of OTUs and ASVs with RDP:

1. Mock: Sanger reference sequences of the mock specimens (18S: *n* = 49, CO1: *n* = 47; mix of short and long fragments for both markers).
2. refDB_mock: Sanger sequence data used in the p‐distance calculations as well as the mock specimens (18S: *n* = 458, CO1: *n* = 311; mix of short and long fragments for both markers).
3. refDB_mock_trimmed: 18S_refDB_mock trimmed to the short amplicon.
4. Silva99_refDB_mock: 20,201 eukaryotic 18S sequences extracted from the Silva database (99% OTUs, release 123 for Qiime1) plus refDB_mock sequences trimmed to the short amplicon region.
5. Silva99_refDB_mock_Nematoda: 2,161 18S nematode sequences extracted from Silva99 plus refDB_mock.

Databases 1–4 were tested in UClust*_ref* while all five were used as training sets in RDP with a confidence estimate of 0.80 to test the effect of sequence abundance, length, and taxonomic specificity, that is, inclusion or exclusion of non‐nematode sequences. Reads from each primer dataset were clustered at 80% and 90%–99% similarity in 1% intervals with singletons discarded. Data were rarefied to the lowest number of observations to obtain α‐diversity metrics (Shannon/Simpson indices, observed OTUs) using the single_rarefaction.py and alpha_rarefaction.py scripts in Qiime1 for the 94%, 97%, and 99% clustering threshold. The effect of experimental treatment (equimolar vs. isovolumetric) was tested for statistical significance [distance metric: weighted UniFrac (Lozupone & Knight, 2005), method: PERMANOVA, 999 permutations] using the beta_diversity_though_plots.py and compare_categories.py scripts in Qiime1 at 94% and 99% similarity. For the Dada2 pipeline, gene‐specific adapter sequences were removed from forward and reverse reads using Cutadapt as previously described (not anchored), fed into RStudio (Version 1.1.423), and processed following the suggested tutorial (https://benjjneb.github.io/dada2/tutorial.html) with the exception of the quality filter parameters which were as follows: maxN=0, maxEE=c(2,5), rm.phix=TRUE, and truncQ=2. Taxonomic assignment with RDP was completed in Qiime1; all results were graphed in GraphPad; bioinformatic commands are provided in Supporting Information S3. Neighbor‐joining trees of the 18S refDB sequences were made in MegaX (model: p‐distance) based on a Muscle alignment in Geneious with default parameters.

### 2.5 Environmental sample

The bioinformatic pipeline which generated the closest resemblance to the taxonomic diversity present in the mock community was applied to three environmental samples (A2–A4) from the intertidal flat of the Can Gio Biosphere Reserve in southern Vietnam which is part of a different study investigating nematode diversity in relation to anthropogenic disturbance. These samples were identified morphologically, thus providing an optimal trial of our pipeline. Sediment was collected using a 10 cm^2^ core of which the top 2 cm was pooled from three replicate cores, homogenized, and again divided into three parts kept frozen in liquid nitrogen, formalin, or DESS. Nematodes were extracted using Ludox density gradient centrifugation (Burgess, 2001) and preserved in DESS (Yoder, Ley, & King, 2006). Samples were poured over a 38‐µm sieve and washed with water before DNA extraction. The collected wash‐off was centrifuged at 1905 RCF for 15 min, after which the supernatant was removed. DNA extraction was completed as described in Derycke, Sheibani Tezerji, Rigaux, and Moens (2012). Library preparation, sequencing, and bioinformatic analyses were completed as described above for the mock communities. For each formalin‐fixed sample, nematodes were counted. Morphological identification was performed for 200 specimens, after which the obtained species counts were used for the per sample nematode species abundances.

## 3 RESULTS

### 3.1 Sanger data reference database

**Figure 1:**
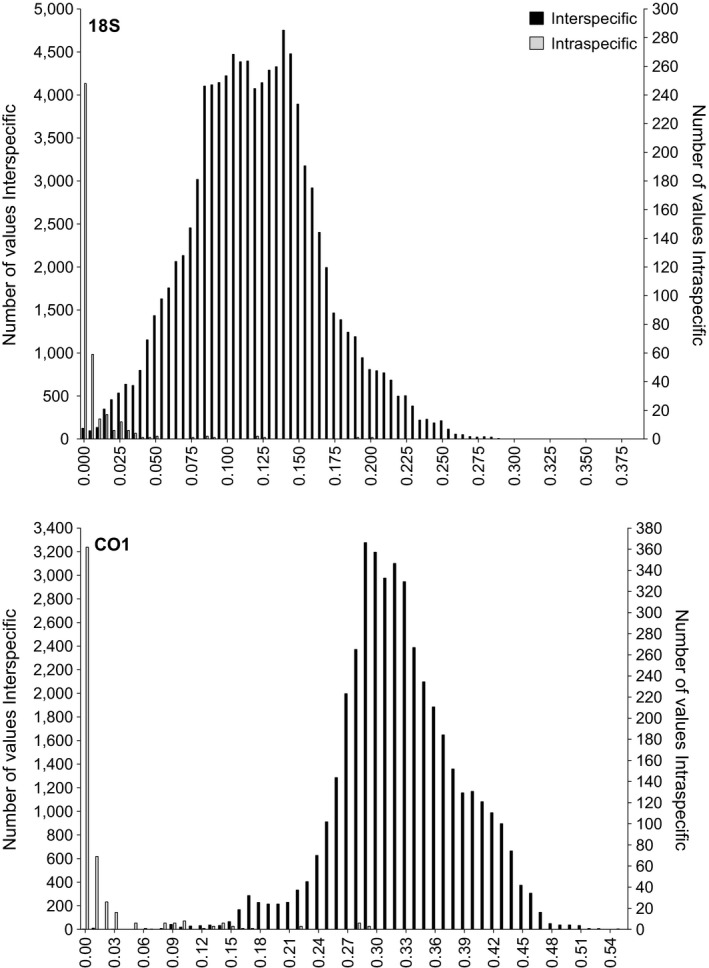
Frequency and distribution of p‐distance values derived from the ribosomal (top) and mitochondrial (bottom) reference sequence databases used in this study. 18S: interspecific [0–0.381], mean = 0.121, *SD* = 0.045 | intraspecific [0–0.199], mean = 0.007, *SD* = 0.021; CO1: interspecific [0–0.546], mean = 0.324, *SD* = 0.062 | intraspecific [0–0.288], mean = 0.018, *SD* = 0.048

The database consisted of 438 ribosomal and 290 mitochondrial sequences, collectively representing 9 out of 25 nematode orders included in the World Register of Marine Species (WoRMS), 116 genera and 274 species (18S: 244 spp., CO1: 154 spp.; congenerics with identical sequences included in S4). The alignment length was 654 bp and 426 bp for 18S and CO1 containing 16 and three indels, respectively. The 18S interspecific p‐distances were distributed between 0 and 0.381 and overlapped with intraspecific p‐distances which ranged from 0 to 0.199. The mitochondrial data were more segregated than the 18S data; intraspecific distances ranged from 0 to 0.288 and interspecific between 0 and 0.546 (Figure 1). Species *Microlaimus honestus *and *Robbea porosum* were characterized by high intraspecific diversity with p–distance values exceeding 0.20 and 0.25, respectively. Within the intraspecific fraction, 65% and 69% of values were equal to zero for 18S and CO1, respectively. The ad hoc species delineation threshold for CO1 was 0.0664, which was approximated to 94% similarity while the average threshold for the six families in 18S was 0.0148, approximated to OTU clustering at 99% (the median was used in the case of intervals, Table 1). Instances of interspecific p‐distances less than 0.01 and 0.06 were typically associated with cryptic species complexes (Supporting Information Figure S8 and S9).

**Table 1:** Threshold distance and relative error by family for 18S reference sequences, median value in brackets

Family	Threshold distance	Relative error
Chromadoridae	0.0023	0.2000
Cyatholaimidae	0.0002	0.0500
Oncholaimidae	0.0051–0.0120 (0.0085)	0.0869
Oxystominidae	0.0051	0.0500
Sphaerolaimidae	0.0619	0.0500
Xyalidae	0.0089–0.0133 (0.0111)	0.1143

### 3.2 Bioinformatics

#### 3.2.1 Read assembly, quality check, and similarity thresholds

**Figure 2:**
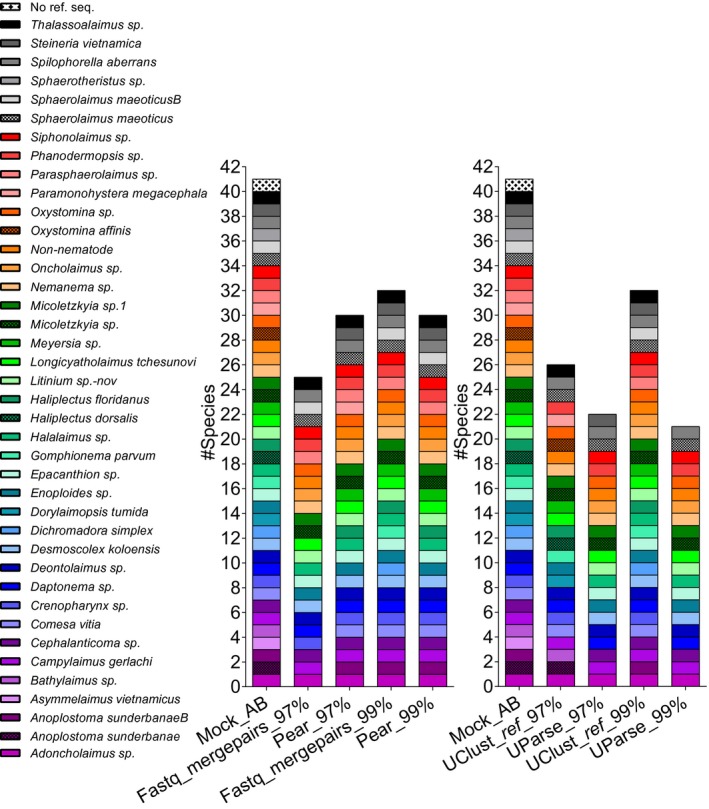
Impact of read‐merging strategy, clustering algorithms, and similarity threshold on taxonomic composition. Left: UClust_ref OTUs for Pear and Fastq_mergepairs reads; right: UClust_ref and UParse OTUs for Fastq_mergepairs reads at 97% and 99% similarity. RDP training set: mock, UClust_ref database: Silva99_refDB_mock, and Mock_AB: true mock composition

Pear and Fastq_mergepairs assembled 98% and 59% of reads across the three primer pairs, of which 69% and 88% were retained by the Fastq_filter, respectively. Forward and reverse primers were removed in 68% and 53% of reads for Pear and Fastq_mergepairs. The two chimera‐checking algorithms flagged 9.92% and 14.47% of reads in the 18S data for Pear and Fastq_mergepairs. The JB2 and JB3 data combined contained fewer chimeras than 18S: 0.12% in Pear reads and 0.11% in Fastq_mergepairs while 3% of CO1 reads were removed for Pear and Fastq_mergepairs due to the presence of stop codons. Overall, 67% and 46% of 18S reads and 66% and 55% of CO1 reads were retained when assembled using Pear and Fastq_mergepairs, respectively. Despite a lower percentage of assembled reads, UClust_*ref *18S OTUs resulting from Fastq_mergepairs were assigned to five and two additional species compared to Pear at 97% and 99% similarity, respectively (Figure 2, left); thus, further testing of clustering databases and RDP training sets was focused on the Fastq_mergepairs datasets. In the Dada2 pipeline, 48%, 63%, and 56% of reads passed the quality filter for 18S, JB2, and JB3, respectively; of these 87%, 100%, and 99% were merged. Chimeras accounted for 19%, 13%, and 0.3% of reads; overall 34%, 54%, and 55% of reads passed the pipeline to be assigned to ASVs for 18S, JB2, and JB3, respectively. Stop codons were found in 3/78 and 29/111 ASVs in JB2 and JB3 datasets. All values are averages provided in Supporting Information Table S4.

Similarity threshold strongly influenced the number of species assignments with RDP in both UClust_*ref* and USearh9 18S OTUs (Figure 2, right); six additional species were detected in the former at 99% compared to 97%. The reverse was true for USearch9 in which one species less was assigned at 99%; overall USearch9 OTUs resulted in the least number of assignments and were thus excluded from further taxonomic comparisons.

#### 3.2.2 OTU picking and amplicon sequence variants (ASVs)

**Figure 3:**
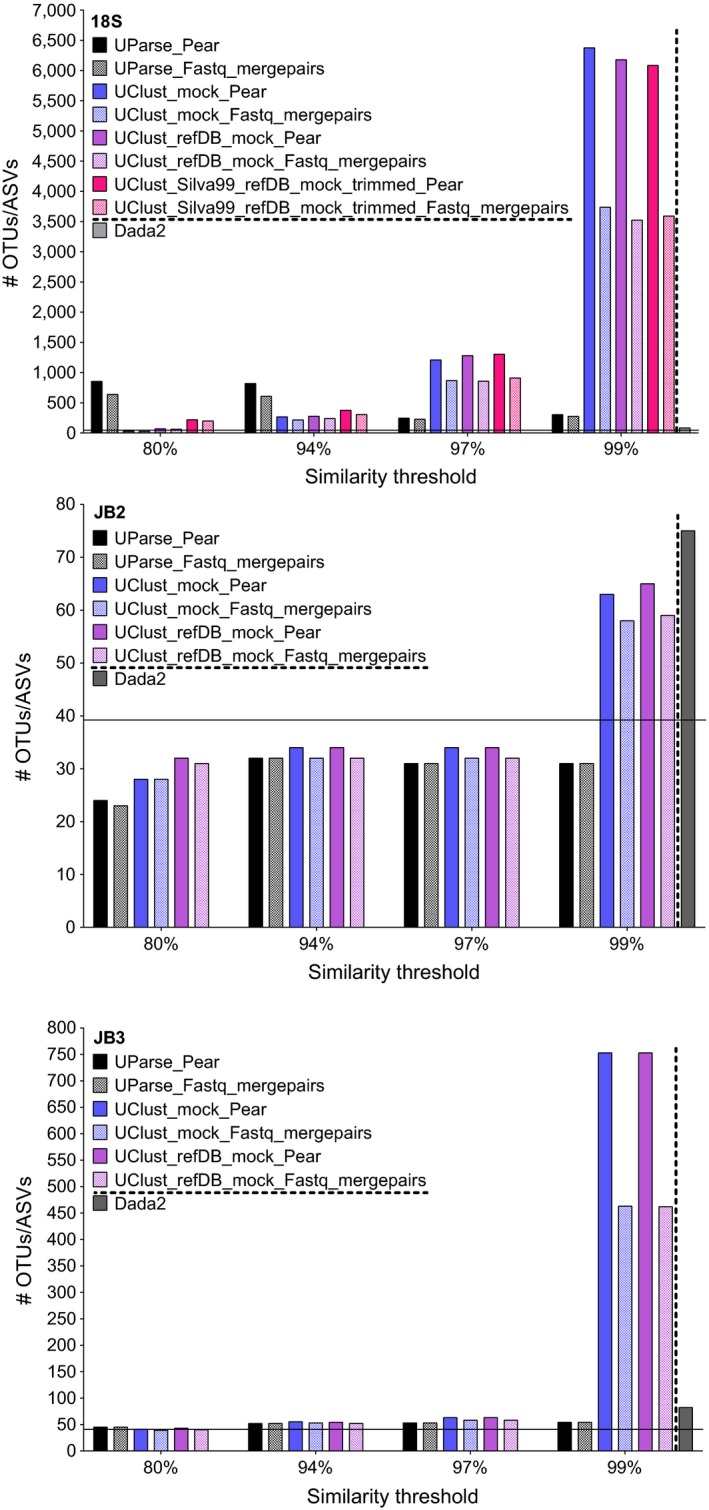
Number of Dada2 ASVs and OTUs for 18S (top), JB2 (middle), JB3 (bottom) reads merged with Pear and Fastq_mergepairs at 80%, 94%, 97%, and 99% similarity with UClust_ref and UParse. UClust_ref databases 18S: mock, refDB_mock, Silva99_refDB_mock, JB2/JB3: mock, refDB_mock. Horizontal bar indicates number of nematode species included in the mock community (*n* = 39)

The two clustering algorithms generated distinct results in the 18S data which differed only in magnitude between Pear and Fastq_mergepair reads (Figure 3, top). UClust*_ref* exhibited an intuitive pattern with number of OTUs increasing with clustering threshold; this was gradual up to 97%, after which point the increase was exponential (Supporting Information Figure S5). The number of UClust*_ref* OTUs was largely similar across reference databases, generating an average of 6,212 and 3,617 OTUs for Pear and Fastq_mergepair reads at 99%, respectively. Clustering at 97% resulted in an average of 1,263 and 878 OTUs for Pear and Fastq_mergepair reads. Counterintuitively, USearch9 generated the highest number of OTUs at the lowest clustering similarity, decreasing substantially thereafter to reach a minimum at 97% (Pear = 245, Fastq_mergepairs = 228), followed by a slight increase at 99% similarity (Pear = 303, Fastq_mergepairs = 275). Dada2 generated the most realistic estimate of species richness with 83 ASVs.

The CO1 data differed from the 18S data as results were strongly similar between the two clustering and read assembly algorithms, and across clustering thresholds excluding 99%. At 99%, the number of JB2 and JB3 UClust*_ref* OTUs increased dramatically yet remained virtually unchanged in USearch9 (Figure 3, middle/bottom). JB2 reads at 94% produced 32 OTUs with USearch9 (Pear=Fastq_mergepairs); UClust*_ref* resulted in 34 (Pear) and 32 (Fastq_mergepairs) OTUs irrespective of reference database. The JB2 dataset yielded fewer OTUs despite having over twice as many reads as JB3. At 94%, JB3 generated 52 OTUs in USearch9 (Pear=Fastq_mergepairs); UClust*_ref* OTUs numbered 55, 53 with the mock reference database and 54, 52 using refDB_mock for Pear and Fastq_mergepair reads, respectively. The Dada2 pipeline resulted in 75 and 82 ASVs for JB2 and JB3, respectively.

#### 3.2.3 Influence of training sets on RDP classifier performance

**Figure 4:**
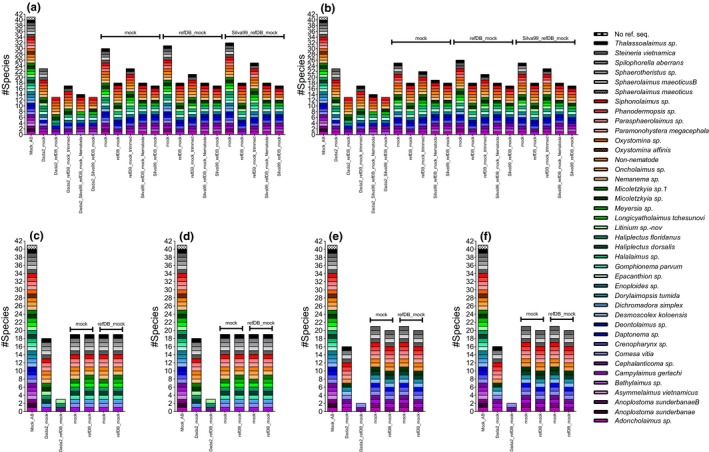
Species taxonomy assigned with the RDP classifier to Dada2 ASVs and UClust_ref OTUs. Top: 18S at 99% (a) and 97% similarity (b); bottom: JB2 at 94% (c) and 97% similarity (d), JB3 at 94% (e) and 97% (f) similarity. Mock_AB: true mock composition. Above bars: UClust_ref database, X‐axis: RDP training set

The RDP classifier was tested with progressively larger training sets; taxonomic assignment of the ribosomal data with the mock training set yielded the highest number of genera and species identifications (30/35, 86%; 32/39, 82%, respectively) in Fastq_mergepairs reads and UClust*_ref* OTUs at a clustering threshold of 99% (Figure 4a, Supporting Information Figure S6A). ASVs were assigned to 23/39 (59%) species. Taxonomic diversity represented in the training set strongly influenced the performance of RDP; regardless of clustering reference database or clustering threshold (97%/99%) used for Uclust_*ref* OTUs, the smallest taxon‐specific training set (mock) yielded the highest number of genus and species identifications throughout. The Silva99_refDB_mock training set containing 20,201 eukaryotic sequences of which 2,161 are nematodes resulted in the least number of genera and species identified at both 97% and 99% similarity as well as the ASVs (Figure 4a,b, Supporting Information Figure S6A,B). The exclusion of non‐nematode sequences (i.e., Silva99_refDB_mock_Nematoda) recovered one additional species at both 97% and 99% similarity compared to the Silva99_refDB_mock training set. Apart from taxonomic composition, trimming reference sequences to the shorter amplicon improved taxonomic assignment by RDP, detecting 4–6 additional species compared to full‐length sequences at both 97% and 99% similarity OTUs and ASVs.

The number of species and genera detected in JB2 mitochondrial OTUs and ASVs was comparable between algorithms and identical across training sets at 94% and 97% similarity (Figure 4c,d, Supporting Information Figure S6C,D). Similarly, the JB3 OTU assignments were identical at 94% and 97%, with the mock training set gaining one additional species over refDB_mock (Figure 4d,e, Supporting Information Figure S6E,D). Species detections were equal between training sets for JB2 OTUs (19/39, 49%) with 18 shared species while *Oncholaimus* sp. and *Meyersia* sp. were exclusive to mock and refDB_mock training sets, respectively; JB3 OTUs were assigned to a maximum of 21/39 (54%) species with the mock training set detecting one additional species (*Adoncholaimus* sp.) compared to refDB_mock. The ASVs detected a similar number of species as the OTUs; 18/39 (46%) and 16/39 (41%) for JB2 and JB3 reads, respectively, with the mock training set. Assignments with refDB_mock were strikingly reduced with just two and three species for JB2 and JB3 ASVs.

#### 3.2.4 Taxonomic assignment of OTUs/ASVs across markers

**Figure 5:**
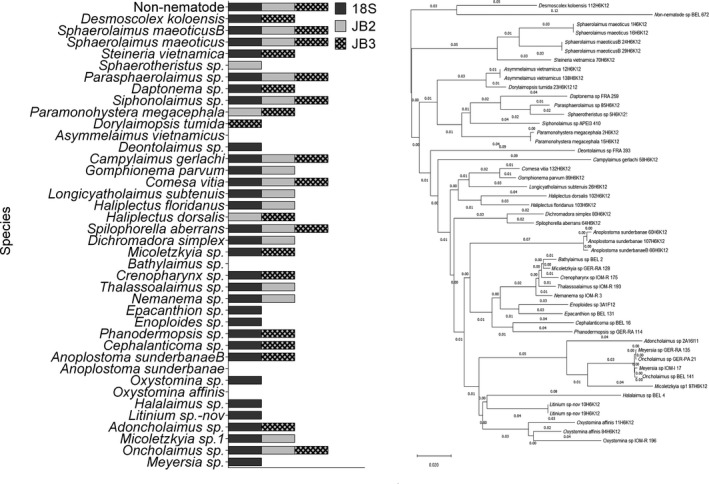
Species taxonomy of UClust_ref OTUs with the RDP classifier for 18S at 99% (dark grey) and JB2/JB3 (light grey/chequered) at 94% (left). Species order based on neighbor‐joining tree of the 18S mock sequences (right; model: p‐distance, bootstraps: 100). RDP training set: mock, UClust_ref database 18S: Silva99_refDB_mock; JB2/JB3: refDB_mock

The combined taxonomic assignment of UClust_*ref* OTUs across the three loci successfully detected 33/35 (94%) genera and 36/40 (92%) species, of which 23 genera and 25 species were shared between 18S and either mitochondrial dataset (Figure 5). Independently, 18S OTUs were assigned to 30/35 genera, 32/39 species and JB2/JB3 OTUs were assigned to 26/35 genera, 29/40 species, respectively. Within the mitochondrial dataset, nine genera and 10 species were detected in the JB3 OTUs exclusively while five genera and eight species were specific to JB2. Detection of the specimens *Sphaerotheristus sp.* and *Dorylaimopsis tumida *was exclusive to the JB2 and JB3 OTUs, respectively, while seven species (*Oxystomina* sp., *Meyersia* sp., *Litinium sp‐nov., Halalaimus* sp., *Epacanthion* sp.,* Enoploides* sp., and *Deontolaimus* sp.) were only detected in 18S OTUs.

The 18S, JB2, and JB3 ASVs collectively detected 29/35 (83%) genera and 30/39 (77%) species of which seven (*Oncholaimus* sp., *Micoletzkyia* sp., *Siphonolaimus* sp., *Sphaerolaimus maeoticus, Sphaerolaimus maeoticusB, Campylaimus gerlachi*, and *Spilophorella aberrans*) were shared. Six unique species were detected in ribosomal ASVs (*Epacanthion* sp., *Oxystomina* sp., *Daptonema* sp., *Halalaimus* sp., *Steineria vietnamica*, and *Enoploides* sp.), three in JB2 (*Haliplectus dorsalis, Dichromadora simplex*, and *Sphaerotheristus* sp.) while *Dorylaimopsis tumida* was exclusive to JB3.

#### 3.2.5 Taxon abundance

**Figure 6:**
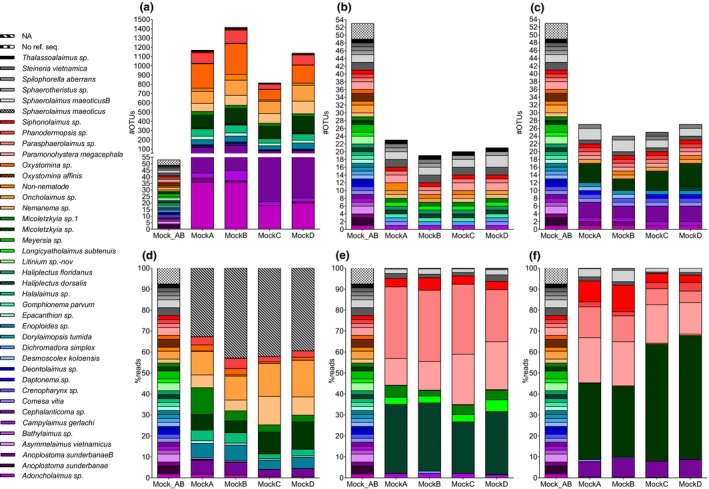
Species taxonomy with the RDP classifier to UClust_ref OTUs (top) and percent reads (bottom) for 18S (a,d), JB2 (b,e), and JB3 (c,f). RDP training set: mock, UClust_ref database 18S: Silva99_refDB_mock, JB2/JB3: refDB_mock, NA = no assignment to species level. Mock_AB: true mock composition

**Figure 7:**
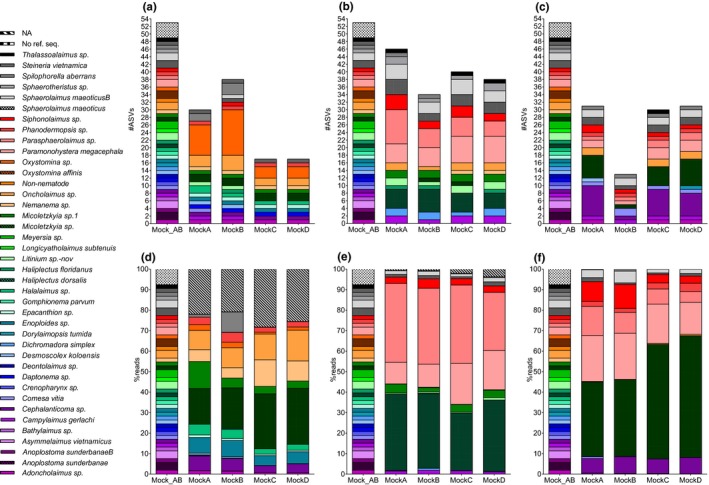
Species taxonomy with the RDP classifier to Dada2 ASVs (top) and percent reads (bottom) for 18S (a,d), JB2 (b,e), and JB3 (c,f). RDP training set: mock, NA = no assignment to species level. Mock_AB: true mock composition

The number of OTUs per species was variable in the 18S dataset; *Oxystomina* sp., *Micoletzkyia* sp., and *Oncholaimus* sp. were overrepresented with an average of 225, 151, and 149 OTUs per mock, respectively (Figure 6a). These taxa were assigned to two to five highly abundant OTUs (1,000–69,000 reads) and several hundred rare OTUs (<800 reads). The ASVs exhibited a similar pattern, most evident for *Oxystomina* sp. which was assigned to 16 ASVs, four of which represented between 200 and 5,000 reads, while the remaining 12 consisted of less than 20 (Figure 7a). Interestingly, dominance in terms of OTUs/ASVs was not mirrored in reads for *Oxystomina sp*. which on average represented ca. 2% of reads (Figures 6d, 7d).

OTU abundance in the mitochondrial datasets was concordant with the known composition of the mocks yet variable in ASVs (Figures 6b,c, 7b,c). The maximum number of OTUs per species was three for JB2 (*Sphaerolaimus maeoticusB*) and six for JB3 (*Micoletzkyia* sp.), while nine (*Parasphaerolaimus* sp., *Paramonohystera megacephala*) and 18 (*Cephalanticoma* sp.) ASVs were found in JB2 and JB3, respectively. Similar to the 18S data, the majority of mitochondrial ASVs per species were very rare; the four most abundant ASVs represented over 98% of reads for *Parasphaerolaimus* sp., *Paramonohystera megacephala,* and *Cephalanticoma* sp. in JB2 and JB3. The most abundant species in terms of reads were *Parasphaerolaimus* sp. in JB2 and *Micoletzkyia* sp. in JB3 OTUs and ASVs (Figure 6e,f, 7e,d).

### 3.3 Equimolar versus isovolumetric pooling

The taxonomic assignment of OTUs was comparable between equimolar and isovolumetric libraries with no statistically significant differences using weighted UniFrac distance (*p*‐values: 18S:.0.342; JB2: 0.330; JB3: 0.657). Equimolar pooling did result in slightly higher diversity estimates for 18S and JB3 (Supporting Information Figure S3A–C, G‐I). Rarefaction indicated that the number of OTUs in the 18S dataset was far from saturated, while these plateaued in the mitochondrial datasets (Supporting Information Figure S3D–I). Indices of diversity in the 18S dataset closely resembled that of the mock community, where MockA (5.36) slightly exceeded and MockB (5.15) reached 98% of the true Shannon value. The CO1 data were consistently less diverse with Shannon and Simpson indices remaining below 2.80 and 0.82, respectively, for both JB2 and JB3 reads.

### 3.4 Environmental sample

**Figure 8:**
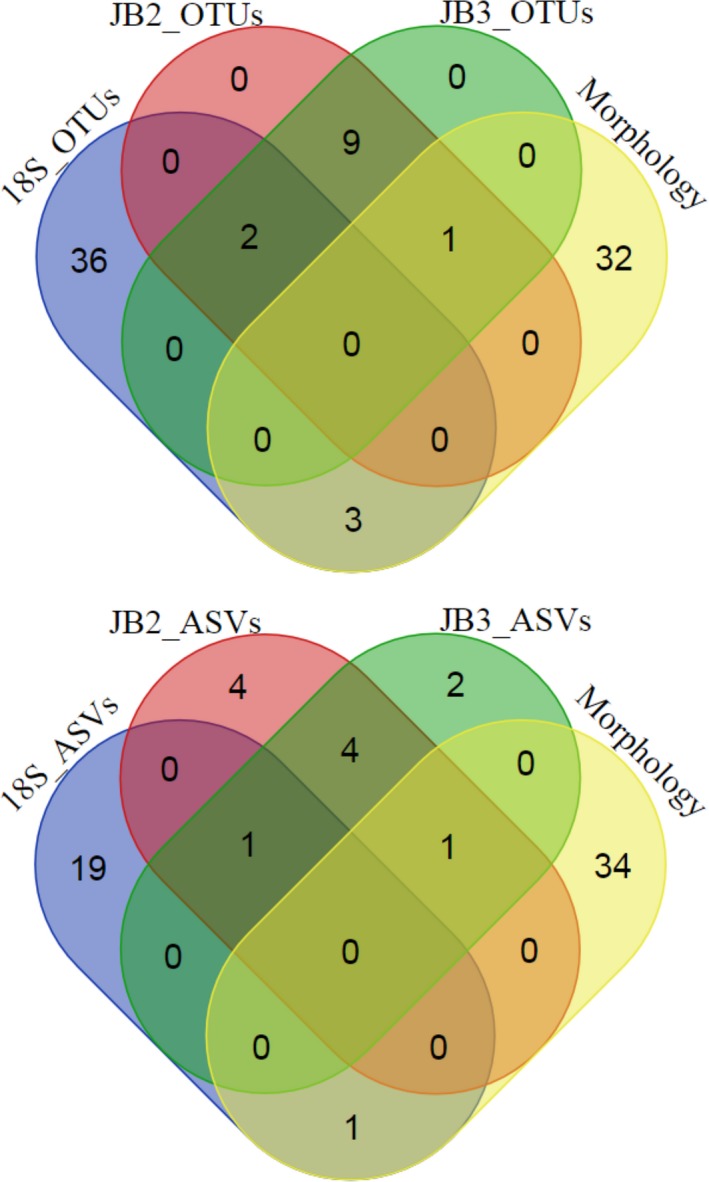
Venn diagrams of morphologically identified nematode species and best performing RDP training sets for 18S, JB2, and JB3 OTUs (top) and ASVs (bottom)

Thirty‐six nematode species were identified morphologically across all three samples. Reads were merged with Fastq_mergepairs and clustered at 99% and 94% using the Silva99_refDB_mock and refDB_mock databases for 18S and JB2/JB3, respectively. UClust_*ref* generated 15,872, 405, 186 OTUs and Dada2 264, 283 and 282 ASVs for 18S, JB2, and JB3, respectively. Two ASVs in the JB3 dataset contained stop codons. OTUs were assigned to a maximum of 41, 12, and 12 nematode species while ASVs to 21, 10, and 8 for 18S, JB2, and JB3, respectively. Morphological and molecular identifications were nearly mutually exclusive in all instances (Figure 8); no species were shared between morphology and mitochondrial OTUs or ASVs. In contrast to 18S mock OTUs/ASVs, using the smallest training set for taxonomic assignment generated the fewest species detections (OTUs: 11, ASVs: 13) while Silva99_refDB_Nematoda and Silva99_refDB_mock identified the highest number of nematode species (OTUs: 41, ASVs: 21, respectively). A larger training set detected between one and three additional species detections for JB2 and JB3 OTUs/ASVs.

## 4 DISCUSSION

### 4.1 Species‐level identification using curated 18S and CO1 Sanger sequence data

The reference sequence databases identified 99% and 94% as the clustering threshold for nematode species delimitation for 18S and CO1 data, respectively. For 18S, 99% similarity has independently been shown to be the most appropriate threshold for identifying nematode communities (Dell'Anno, Carugati, Corinaldesi, Riccioni, & Danovaro, 2015; Porazinska, Giblin‐Davis, Sung, et al., 2010) as well as other small marine metazoans (Brown, Chain, Crease, MacIsaac, & Cristescu, 2015). Taxonomic assignment of the 99% OTUs resulted in the most accurate species‐level taxonomic representation of the mock community. For the mitochondrial OTUs, species detections were equivalent between 94% and 97% similarity. Moreover, species‐level assignments in 18S OTUs were more abundant than for CO1 despite limitations of the former marker due to the absence of a definite barcoding gap [Figure 1, (Armenteros et al., 2014; Derycke, Vanaverbeke, et al., 2010)]. This likely reflects the higher variability at the primer locations of CO1 which hampers amplification of the fragment across the whole phylum (De Ley et al., 2005). Determining species delineation thresholds is challenging given overlapping intra‐/inter‐specific distances within diverse and insufficiently described groups (Meyer & Paulay, 2005); nonetheless, the calculation of empirically derived taxon‐specific clustering thresholds should be adopted over the use of standardized parameters (Alberdi, Aizpurua, Gilbert, & Bohmann, 2018). Our results show that the use of p‐distances from curated reference sequence databases is a promising approach to determine these thresholds and that future metabarcoding studies could benefit greatly from adopting taxon‐specific clustering thresholds as illustrated in the present study as well as Brown et al. (2015).

### 4.2 Clustering open‐reference, denovo, or not at all

Clustering threshold is one of the most important factors influencing OTU composition in heuristic algorithms such as USearch9 and UClust*_ref*/UClust_*denovo* (Schmidt, Matias Rodrigues, & von Mering, 2015). UClust*_ref* and USearch9 produced contrasting OTU abundance patterns in the ribosomal reads; the number of OTUs in the former increased with clustering threshold, whereas in the latter, these followed a decreasing trend from maximum at 80% to minimum at 97%. The number of USearch9 OTUs was approximately five times higher than the taxa included in the mocks, yet over an order of magnitude less than UClust*_ref* OTUs. USearch9 applies *denovo* chimera detection concurrent to OTU picking, which effectively alters the collection of reads being clustered at any given threshold (Schmidt et al., 2015). The inflated estimation of OTU abundance with UClust*_ref* only in the 18S data can thus be justified in two ways: First, UClust*_denovo* tends to “oversplit” sequence data (Schmidt et al., 2015), and this is likely to be true also for UClust*_ref* given that the two differ only with respect to what collection of sequences reads are being compared to; second, when looking at OTUs assigned to a particular species, these typically consist of one or few abundant OTUs representing the majority of reads (“head”) and a large collection of rare variants typically containing very few reads or singletons (“tail”). These are thought to represent intraspecific variation associated with ribosomal tandem repeats which are largely responsible for inflated OTU abundance and can be species‐specific (Porazinska, Giblin‐Davis, Sung, et al., 2010). The number of “tail” OTUs (1–480, Figure 6) was considerably higher than the 3–28 range reported in Porazinska, Giblin‐Davis, Esquivel, et al. (2010) while ASVs were more similar with 1–16 per species (Figure 7). A large number of OTUs need not be reflected in the respective amount of reads as the vast majority would be rare. A strong disagreement in relative abundance of OTUs and reads was observed in *Oxystomina* sp., suggesting this species exhibits increased intragenomic variation. The “head‐tail” pattern was far less prevalent for CO1, where just three species contained more than one OTU. Moreover, the total number of OTUs per species was substantially reduced (3–6), suggesting that intra‐individual variation is much lower for CO1; given the protein‐encoding function of the gene, this may be the consequence of purifying selection.

Heuristics such as USearch9 and UClust_*ref* have been shown to outperform hierarchical methods due to reduced computational requirements; USearch9 in particular has been recommended due to increased accuracy in OTU‐based diversity estimates (Flynn, Brown, Chain, MacIsaac, & Cristescu, 2015). Moreover, *denovo* clustering as applied in USearch9 has been advised over reference‐based clustering due to improved OTU stability (Westcott & Schloss, 2015), while He et al. (2015) concluded that this is inherent to both hierarchical and heuristic methods, regardless of whether *denovo* or reference‐based is implemented in the latter. Concurrently, it may be possible to circumvent a lack of congruence across different methods and algorithms by thorough and stringent quality filtering prior to OUT picking (May, Abeln, Crielaard, Heringa, & Brandt, 2014). Identification of 92% of mock species across primers in UClust_*ref* OTUs suggests our quality filtering was indeed effective and that any dissimilarities to USearch9 must be attributable to the algorithm itself.

The detection of nematode species was greatly improved using UClust*_ref* in combination with a high‐quality reference database compared to USearch9 (Figure 2). Furthermore, open‐reference clustering links sequence reads to known biological entities, does not discard any data, and requires reduced computational capacity (Navas‐Molina et al, 2013). The diversity and taxon specificity of reference database sequences had a negligible effect on the number of ribosomal and mitochondrial OTUs generated by UClust*_ref,* and subsequent taxonomic assignment was strongly similar between the different OTU sets for both markers. Ultimately, Silva99_refDB_mock yielded the most accurate taxonomic description of the mock community suggesting that one should seek the largest and most diverse reference database for open‐reference‐based OUT picking.

The cluster‐free Dada2 pipeline produced the most realistic estimates of species richness yet taxonomic assignment of ASVs in both ribosomal and mitochondrial datasets were reduced compared to UClust_*ref* OTUs, collectively detecting 6/39 (15%) fewer species. These assignments, however, were far less redundant across markers with just seven species shared between ribosomal and mitochondrial ASVs compared to 25 in OTUs. Moreover, given their inherent reproducibility, comparability across datasets (Callahan, McMurdie, & Holmes, 2017), and ease of executing the pipeline, ASVs provide a competitive alternative to OTUs.

### 4.3 Effect of training sets on taxonomic assignment with RDP

The number of taxonomic assignments in ribosomal OTUs differed substantially between training sets and highlighted the influence of sequence homology as well as diversity. Similar to 16S bacterial data (Werner et al., 2012), trimming sequences to the shorter amplicon, and thus increasing sequence homology, improved the performance of RDP in ribosomal OTUs and Dada2 ASVs. Taxonomic assignment of OTUs and ASVs was substantially improved with the smallest nematode‐exclusive training set 18S_mock (<50 sequences) compared to a larger one such as Silva99_refDB_mock (ca. 2,100 sequences) or the most extensive training set Silva99_refDB_mock (>20,000 sequences) including representatives across the eukaryotic domain. Concurrently, diverse, taxon‐specific training sets such as Silva99_refDB_mock and Silva99_refDB_mock_Nematoda detected the most nematode species in the Vietnam sample suggesting that similarity between sample and training set in terms of diversity is an important factor influencing the accuracy of the RDP classifier. The use of arbitrarily large databases has been shown to hinder resolution to genus/species level due to increased competition in the search space (Ritari et al., 2015). This effect was most prominent in RDP assignments to mitochondrial ASVs with the refDB_mock training set, presumably indicating a shortcoming of ASVs in highly variable functional gene loci. The inclusion of superfluous sequences was more impactful than that of non‐homologous regions, indicating that in order to maximize the accurate identification of diversity, RDP training sets should be taxon and region specific with sequences truncated to the length of the amplified fragment of interest.

### 4.4 Moving forward in marine nematode metabarcoding

Although a 1:1 correspondence between OTUs and number of taxa has been reported in some mock communities (Brown et al., 2015), the expectation thereof may be unrealistic when investigating biological samples with the 18S gene specifically. Taxonomic assignment of OTUs is optional and ultimately at the researchers’ discretion, we nonetheless find it a highly informative component of the bioinformatic pipeline and necessary for the ecological interpretation of HTS data in environmental samples. Given that OTUs clustered at different similarities are not comparable, their abundance being variable by several orders of magnitude across different methods and the promising alternative of clustering‐free algorithms (e.g., DADA2), taxonomic assignment can be a normalizing factor to divergent outputs (Callahan et al., 2016). Although a large proportion of species detections were shared between ribosomal and mitochondrial OTUs, ASV assignments were far less redundant, demonstrating the benefits of a multilocus approach to metabarcoding. Community composition did not differ statistically between equimolar and isovolumetric DNA pooling, suggesting these to be effectively equivalent. Some caution is nonetheless warranted given the limited replication in our data (*n* = 2). In view of the slightly higher alpha diversity estimates obtained with equimolar pooling, we recommend this approach.

## 5 CONCLUSION

The importance of reference sequences coupled to reliable taxonomic labels cannot be overstated as this information is essential for linking taxa present in a sample to the wealth of relevant biological knowledge. Although identification at higher taxonomic levels is relatively straightforward and commonplace (Brannock & Halanych 2015; Cowart et al. 2015; Leray & Knowlton 2015; Capra et al. 2016; Sinniger et al. 2016), species‐level resolution is hindered by the combined effect of incomplete databases, inaccurate designations and the restricted amount of information contained in short sequences generated by HTS platforms (Somervuo et al., 2017). The establishment of HTS technologies in the realm of biology and associated complications for reliable species identification highlight the urgency of creating high‐quality taxon‐specific reference sequence databases such as was implemented in the current study. We have shown that the current state of our database can significantly aid diversity estimates of marine nematodes. The scant availability of nematode reference sequences from diverse habitats such as the deep‐sea, seagrass beds, and tropical coral reefs, however, hinders a comprehensive characterization of novel ecosystems. As such, investing in barcoding initiatives for understudied environments in particular will always be necessary to expand database coverage.

## CONFLICT OF INTEREST

The authors declare no conflict of interest.

## AUTHOR CONTRIBUTIONS

SD, KG, and BT conceived and designed the study. DN, PN, MA, and FB contributed Sanger sequences. KG, FN, AR, and BT curated the data. LM performed the experiments, analysis/interpretation of the data and composed the manuscript with substantial contributions from SD, BT, and AV.

## Data Availability

Sanger data CO1: GenBank accession numbers MG659557‐MG659594. Sanger data 18S: GenBank accession numbers MG669659‐MG670092. HTS reads: NIH Sequence Read Archive BioProject PRJNA420028: Nematoda mock community metabarcoding. Bioinformatic scripts: Supporting Information.
