# Supplementary material for: Metabarcoding free‐living marine nematodes using curated 18S and CO1 reference sequence databases for species‐level taxonomic assignments
Source: Ecol Evol. 2019 Jan 22;9(3):1211–26. doi: 10.1002/ece3.4814 (PMC6374678; doi:10.1002/ece3.4814)
Supplement: Supplementary file 1 [file ECE3-9-1211-s001.docx]

**Supplemental Information for:**

**Metabarcoding free-living marine nematodes using curated 18S and CO1 reference sequence databases for species level taxonomic assignments**

Lara Macheriotou, Katja Guilini, Tania Nara Bezerra, Bjorn Tytgat, Dinh Tu Nguyen, Thi Xuan Phuong Nguyen, Maickel Armenteros, Fehmi Boufahja, Annelien Rigaux, Ann Vanreusel, Sofie Derycke

**Table of Contents:**

| **S1: Source locations of mock community specimens** | Page 2 |
| --- | --- |
| **S2: Bio-informatic pipeline scheme** | Page 3 |
| **S3: Bio-informatic analysis scripts** | Page 4-6 |
| **S4: Detailed read statistics** | Page 6 |
| **S5: Dada2 ASVs and all clustering percentages OTUs** | Page 7 |
| **S6: Genus-level taxonomy ASVs and UClust OTUs 18S, JB2, JB3** | Page 8 |
| **S7: Diversity metrics** | Page 9 |
| **S8: 18S_refDB Neighbor-joining tree** | External file (too large) |
| **S9: CO1_refDB Neighbor-joining tree** | External file (too large) |

Table 1.Mock community composition and specimen source location. ^*^No reference sequence available + not included in C/D, ^**^not included in C/D (insufficient extract), ^***^no CO1 reference sequence.

| **Voucher code** | **18S_Mock** | | **CO1_Mock** | | **Location** | **Zone** |
| --- | --- | --- | --- | --- | --- | --- |
|  | **A/B** | **C/D** | **A/B** | **C/D** |  |  |
| Adoncholaimus_sp_2A16I11 | x | x | x | x | Netherlands | Intertidal |
| Anoplostoma_sp_4C9B12^*^ | x | --- | x | --- | Netherlands | Intertidal |
| Anoplostoma_sunderbanae_107H6K12 | x | x | x | x | Southern Vietnam | Intertidal |
| Anoplostoma_sunderbanae_60H6K12 | x | x | x | x | Southern Vietnam | Intertidal |
| Anoplostoma_sunderbanaeB_66H6K12 | x | x | x | x | Southern Vietnam | Intertidal |
| Asymmelaimus_vietnamicus_12H6K12 | x | x | x | x | Southern Vietnam | Intertidal |
| Asymmelaimus_vietnamicus_138H6K12 | x | x | x | x | Southern Vietnam | Intertidal |
| Bathylaimus_sp_BEL_2 | x | x | x | x | Equatorial North Pacific | Abyssal |
| Campylaimus_gerlachi_58H6K12 | x | x | x | x | Southern Vietnam | Intertidal |
| Cephalanticoma_sp_BEL_16 | x | x | x | x | Equatorial North Pacific | Abyssal |
| Comesa_vitia_132H6K12 | x | x | x | x | Southern Vietnam | Intertidal |
| Crenopharynx_sp_IOM-R_175 | x | x | x | x | Equatorial North Pacific | Abyssal |
| Daptonema_sp_FRA_259 | x | x | x | x | Equatorial North Pacific | Abyssal |
| Deontolaimus_sp_FRA_393 | x | x | x | x | Equatorial North Pacific | Abyssal |
| Desmoscolex_koloensis_112H6K12 | x | x | x | x | Southern Vietnam | Intertidal |
| Dichromadora_simplex_80H6K12 | x | x | x | x | Southern Vietnam | Intertidal |
| Dorylaimopsis_tumida_23H6K12 | x | x | x | x | Southern Vietnam | Intertidal |
| Enoploides_sp_3A1F12 | x | x | x | x | Netherlands | Intertidal |
| Epacanthion_sp_BEL_131 | x | x | x | x | Equatorial North Pacific | Abyssal |
| Gomphionema_parvam_89H6K12 | x | x | x | x | Southern Vietnam | Intertidal |
| Halalaimus_sp_BEL_4^**^ | x | x | x | x | Equatorial North Pacific | Abyssal |
| Haliplectus_dorsalis_102H6K12 | x | x | x | x | Southern Vietnam | Intertidal |
| Haliplectus_floridanus_103H6K12 | x | x | x | x | Southern Vietnam | Intertidal |
| Litinium_sp-nov_10H6K12 | x | x | x | x | Southern Vietnam | Intertidal |
| Litinium_sp-nov_19H6K12 | x | x | x | x | Southern Vietnam | Intertidal |
| Longicyatholaimus_tchesunovi_26H6K12 | x | x | x | x | Southern Vietnam | Intertidal |
| Meyersia_sp_GER-RA_135 | x | x | x | x | Equatorial North Pacific | Abyssal |
| Micoletzkyia_sp_97H6K12 | x | x | x | x | Southern Vietnam | Intertidal |
| Micoletzkyia_sp_GER-RA_128 | x | x | x | x | Equatorial North Pacific | Abyssal |
| Nemanema_sp_IOM-R_3 | x | x | x | x | Equatorial North Pacific | Abyssal |
| Odontophora_sp_8C10B12^*^ | x | --- | x | --- | Netherlands | Intertidal |
| Oncholaimus_sp_BEL_141 | x | x | x | x | Equatorial North Pacific | Abyssal |
| Oncholaimus_sp_GER-PA_21 | x | x | x | x | Equatorial North Pacific | Abyssal |
| Non-nematode (Fungi) | x | x | x | x | ---------- | ---------- |
| Oxystomina_affinis_11H6K12 | x | x | x | x | Southern Vietnam | Intertidal |
| Oxystomina_affinis_84H6K12 | x | x | x | x | Southern Vietnam | Intertidal |
| Oxystomina_sp_IOM-R_196 | x | x | x | x | Equatorial North Pacific | Abyssal |
| Paramonohystera_megacephala_15H6K12 | x | x | x | x | Southern Vietnam | Intertidal |
| Paramonohystera_megacephala_2H6K12 | x | x | x | x | Southern Vietnam | Intertidal |
| Parasphaerolaimus_sp_85H6K12 | x | x | x | x | Southern Vietnam | Intertidal |
| Phanodermopsis_sp_GER-RA_114 | x | x | x | x | Equatorial North Pacific | Abyssal |
| Siphonolaimus_sp_APEI3_410 | x | x | x | x | Equatorial North Pacific | Abyssal |
| Sphaerolaimus_maeoticus_16H6K12 | x | x | x | x | Southern Vietnam | Intertidal |
| Sphaerolaimus_maeoticus_1H6K12 | x | x | x | x | Southern Vietnam | Intertidal |
| Sphaerolaimus_maeoticusB_24H6K12 | x | x | x | x | Southern Vietnam | Intertidal |
| Sphaerolaimus_maeoticusB_29H6K12 | x | x | x | x | Southern Vietnam | Intertidal |
| Sphaerolaimus_sp_1C9B12^*^ | x | --- | x | --- | Netherlands | Intertidal |
| Sphaerotheristus_sp_5H6K12 | x | x | x | x | Southern Vietnam | Intertidal |
| Spilophorella_aberrans_64H6K12^*^ | x | --- | x | --- | Southern Vietnam | Intertidal |
| Spirinia_sp_15C13B12^*^ | x | --- | x | --- | Netherlands | Intertidal |
| Steineria_vietnamica_70H6K12 | x | x | x | x | Southern Vietnam | Intertidal |
| Thalassolaimus_sp_IOM-R_193 | x | x | x | x | Equatorial North Pacific | Abyssal |
| Meyersia_sp_IOM-I_17 | x | x | x | x | Equatorial North Pacific | Abyssal |
| **Total DNA extracts** | 53 | 48 | 53 | 48 |  | |
| **Total Nematoda species** | 39 | 35 | 39 | 35 |  |  |
| **Total reference sequences** | 49 | 48 | 47 | 47 |  |  |

**Read Assembly**pear –f /media/sf_G_DRIVE/NGS_Mock_data/Mocks/Run2_16_12_16/JB3_A/161216_M01270_0259_000000000-ARU1U_1_NX-P7-004_NX-P5-006_1.fastq –r /media/sf_G_DRIVE/NGS_Mock_data/Mocks/Run2_16_12_16/JB3_A/161216_M01270_0259_000000000-ARU1U_1_NX-P7-004_NX-P5-006_2.fastq –o /media/sf_G_DRIVE/NGS_Mock_data/Mocks/Run2_16_12_16/JB3_A/Pear_merged_PE_quality_filter/JB3_A -m 600 -n 50 -t 200 -q 30 -u 0

usearch9 -fastq_mergepairs /media/sf_G_DRIVE/Mock_MiSeq/Run2_16_12_16/CO1/Raw/JB3_A/*R1.fastq –fastqout /media/sf_G_DRIVE/Mock_MiSeq/Run2_16_12_16/CO1/Usearch9_fastq_mergepairs/JB3_A_merged.fastq -fastq_minmergelen 50 -fastq_maxdiffs 10 -fastq_maxmergelen 600 -fastq_minovlen 10

**Quality filter**usearch9 -fastq_filter /media/sf_G_DRIVE/Mock_MiSeq/Run2_16_12_16/CO1/Pear/JB3_A.assembled.fastq -fastqout /media/sf_G_DRIVE/Mock_MiSeq/Run2_16_12_16/CO1/usearch9_fastq_filter/JB3_A.assembled_filtered.fastq -fastqout_discarded /media/sf_G_DRIVE/Mock_MiSeq/Run2_16_12_16/CO1/usearch9_fastq_filter/discarded/JB3_A.assembled_discarded.fastq -fastq_maxee 0.5 -fastq_minlen 300 -fastq_maxns 1

**Primer removal
|**cutadapt -g JB3=^TGGGCATCCTGAGGTTTAT /media/sf_G_DRIVE/Mock_MiSeq/Run2_16_12_16/CO1/Usearch9_fastq_filter/fastq_mergepairs/JB3_A_merged_filtered.fastq –o /media/sf_G_DRIVE/Mock_MiSeq/Run2_16_12_16/CO1/Cutadapt/run4_mergepairs_filter/JB3_A_f.fasta --untrimmed-output /media/sf_G_DRIVE/Mock_MiSeq/Run2_16_12_16/CO1/Cutadapt/run4_mergepairs_filter/untrimmed/JB3_A_f_untrimmed.fasta -O 5 -e 0.1 -m 1

cutadapt -a JB5=CATTTTCATTATGTTTTAAGTTTAGGTGCT$ /media/sf_G_DRIVE/Mock_MiSeq/Run2_16_12_16/CO1/Cutadapt/run4_mergepairs_filter/JB3_A_f.fasta –o /media/sf_G_DRIVE/Mock_MiSeq/Run2_16_12_16/CO1/Cutadapt/run4_mergepairs_filter/JB3_A_f_r.fasta --untrimmed-output /media/sf_G_DRIVE/Mock_MiSeq/Run2_16_12_16/CO1/Cutadapt/run4_mergepairs_filter/untrimmed/JB3_A_f_r_untrimmed.fasta -O 5 -e 0.1 -m 1

**Chimeric sequences removal**identify_chimeric_seqs.py
–i /media/sf_G_DRIVE/Mock_MiSeq/Run2_16_12_16/CO1/CO1_all_seqs_pear_filter.fasta -o /media/sf_G_DRIVE/Mock_MiSeq/Run2_16_12_16/CO1/Qiime/id_chimeric_seqs_usearch61_denovo_ref/pear -m usearch61 --non_chimeras_retention intersection –r /media/sf_G_DRIVE/Mock_MiSeq/Run2_16_12_16/CO1/CO1_refDB_mock.fasta

filter_fasta.py –f /media/sf_G_DRIVE/Mock_MiSeq/Run2_16_12_16/CO1/CO1_all_seqs_pear_filter.fasta -o /media/sf_G_DRIVE/Mock_MiSeq/Run2_16_12_16/CO1/Qiime/id_chimeric_seqs_usearch61_denovo_ref/pear/CO1_pear_filter_no_chimeras.fasta -s /media/sf_G_DRIVE/Mock_MiSeq/Run2_16_12_16/CO1/Qiime/id_chimeric_seqs_usearch61_denovo_ref/pear/chimeras.txt –n

**Add labels**add_qiime_labels.py -i /media/sf_G_DRIVE/Mock_MiSeq/Run2_16_12_16/CO1/Cutadapt/mergepairs_filter/ -m /media/sf_G_DRIVE/Mock_MiSeq/Run2_16_12_16/CO1/CO1_mock_mapping_file.txt -c InputFileName -o /media/sf_G_DRIVE/Mock_MiSeq/Run2_16_12_16/CO1/CO1_all_seqs_mergepairs_filter.fna -n 1

**Stop Codon Removal**

<https://docs.google.com/document/d/1HoaXzXTIxSn--sleBxcfSn9wmsBP_jl6sHFmfP7_Du8/edit?usp=sharing>

**OTU-picking**
pick_open_reference_otus.py –i /media/sf_G_DRIVE/Mock_MiSeq/Run2_16_12_16/CO1/JB3_mergepairs_filter_no_chimeras_inFrame1_no_SC.fasta -o /media/sf_G_DRIVE/Mock_MiSeq/Run2_16_12_16/CO1/Qiime/uclust/mock/mergepairs/JB3_80%/ -f -m uclust -s 0.01 -r /media/sf_G_DRIVE/Mock_MiSeq/Run2_16_12_16/CO1/Mock_CO1_A_B_unaligned_48.fasta --suppress_align_and_tree -p /media/sf_G_DRIVE/Mock_MiSeq/Scripts_results/Qiime/parameters/mock/uclust_parameters_CO1_mock_80%.txt

usearch9 -cluster_otus /media/sf_G_DRIVE/Mock_MiSeq/Run2_16_12_16/CO1/JB3_uniques_pear_filter_no_chimeras_inFrame1_no_SC.fasta -otu_radius_pct 20.0 -minsize 2 -otus /media/sf_G_DRIVE/Mock_MiSeq/Run2_16_12_16/CO1/Usearch9_otus/JB3_pear_filter_otus_80%.fa –uparseout /media/sf_G_DRIVE/Mock_MiSeq/Run2_16_12_16/CO1/Usearch9_otus/JB3_pear_filter_otus_80%.up

**Alpha rarefaction**

biom summarize-table –i /media/sf_G_DRIVE/Mock_MiSeq/Run2_16_12_16/18S/Qiime/uclust/Silva99_refDB_mock_trimmed/mergepairs/97%/otu_table_mc2_12x_filtered_w_tax.biom

alpha_rarefaction.py –i /media/sf_G_DRIVE/Mock_MiSeq/Run2_16_12_16/18S/Qiime/uclust/Silva99_refDB_mock_trimmed/mergepairs/97%/otu_table_mc2_12x_filtered_w_tax.biom –m /media/sf_G_DRIVE/Mock_MiSeq/Run2_16_12_16/18S/18S_mock_mapping_file.txt \ -p /media/sf_G_DRIVE/Mock_MiSeq/Scripts_results/Qiime/parameters/alpha_rarefaction_parameters.txt –o /media/sf_G_DRIVE/Mock_MiSeq/Run2_16_12_16/18S/Qiime/uclust/Silva99_refDB_mock_trimmed/mergepairs/97%/ -f -e 108864

**Beta diversity**

beta_diversity_through_plots.py –i /media/sf_F_DRIVE/Mock_MiSeq_NGS/Run2_16_12_16/18S/Qiime/uclust/Silva99_refDB_mock_trimmed/mergepairs/99%/otu_table_mc2_12x_filtered_w_tax_rarefied.biom -m /media/sf_F_DRIVE/Mock_MiSeq_NGS/Run2_16_12_16/18S/18S_mock_mapping_file.txt -o /media/sf_F_DRIVE/Mock_MiSeq_NGS/Run2_16_12_16/18S/Qiime/uclust/Silva99_refDB_mock_trimmed/mergepairs/99%/beta_diversity --color_by_all_fields -p /media/sf_F_DRIVE/Mock_MiSeq_NGS/Scripts_results/Qiime/parameters/beta_diversity_parameters_18S_99%.txt

**Statistical testing**

compare_categories.py -i /media/sf_F_DRIVE/Mock_MiSeq_NGS/Run2_16_12_16/18S/Qiime/uclust/Silva99_refDB_mock_trimmed/mergepairs/99%/beta_diversity/dm/unweighted_unifrac_dm.txt --method permanova -m /media/sf_F_DRIVE/Mock_MiSeq_NGS/Run2_16_12_16/18S/18S_mock_mapping_file.txt -c Treatment –o /media/sf_F_DRIVE/Mock_MiSeq_NGS/Run2_16_12_16/18S/Qiime/uclust/Silva99_refDB_mock_trimmed/mergepairs/99%/permanova/

**S4**

https://docs.google.com/spreadsheets/d/1Hs_cYDqPbMvI7Zfzzb3yG_iOg6k6GLIMRdmyFe2U1gU/edit?usp=sharing
